# Computational Models of the Notch Network Elucidate Mechanisms of Context-dependent Signaling

**DOI:** 10.1371/journal.pcbi.1000390

**Published:** 2009-05-22

**Authors:** Smita Agrawal, Colin Archer, David V. Schaffer

**Affiliations:** 1 Department of Chemical Engineering, University of California Berkeley, Berkeley, California, United States of America; 2 Department of Bioengineering, University of California Berkeley, Berkeley, California, United States of America; 3 Helen Wills Neuroscience Institute, University of California Berkeley, Berkeley, California, United States of America; University of Tokyo, Japan

## Abstract

The Notch signaling pathway controls numerous cell fate decisions during development and adulthood through diverse mechanisms. Thus, whereas it functions as an oscillator during somitogenesis, it can mediate an all-or-none cell fate switch to influence pattern formation in various tissues during development. Furthermore, while in some contexts continuous Notch signaling is required, in others a transient Notch signal is sufficient to influence cell fate decisions. However, the signaling mechanisms that underlie these diverse behaviors in different cellular contexts have not been understood. *Notch1* along with two downstream transcription factors *hes1* and *RBP-Jk* forms an intricate network of positive and negative feedback loops, and we have implemented a systems biology approach to computationally study this gene regulation network. Our results indicate that the system exhibits bistability and is capable of switching states at a critical level of Notch signaling initiated by its ligand Delta in a particular range of parameter values. In this mode, transient activation of Delta is also capable of inducing prolonged high expression of Hes1, mimicking the “ON” state depending on the intensity and duration of the signal. Furthermore, this system is highly sensitive to certain model parameters and can transition from functioning as a bistable switch to an oscillator by tuning a single parameter value. This parameter, the transcriptional repression constant of *hes1*, can thus qualitatively govern the behavior of the signaling network. In addition, we find that the system is able to dampen and reduce the effects of biological noise that arise from stochastic effects in gene expression for systems that respond quickly to Notch signaling.

This work thus helps our understanding of an important cell fate control system and begins to elucidate how this context dependent signaling system can be modulated in different cellular settings to exhibit entirely different behaviors.

## Author Summary

The Notch signaling pathway is an evolutionarily conserved signaling system that is involved in various cell fate decisions, both during development of an organism and during adulthood. While the same core circuit functions in various different cellular contexts, it has experimentally been shown to elicit varied behaviors and responses. On the one hand, it functions as a cellular oscillator critical for somitogenesis, whereas in other situations, it can function as a cell fate switch to pattern developing tissue, for example in the *Drosophila* eye. Furthermore, malfunctioning of Notch signaling is implicated in various cancers. To better understand the underlying mechanisms that allow the network to function distinctly in different contexts, we have mathematically modeled the behavior of the Notch network, encompassing the Notch gene along with two of its downstream effector transcription factors, which together form a network of positive and negative feedback loops. Our results indicate that the qualitative and quantitative behavior of the system can readily be tuned based on key parameters to reflect its multiple roles. Furthermore, our results provide insights into alterations in the signaling system that lead to malfunction and hence disease, which could be used to identify potential drug targets for therapy.

## Introduction

Cells continuously receive signals from their microenvironments – including factors present in the extracellular matrix, soluble media, and surrounding cells – which collectively influence cell function and behavior via activating intracellular signal transduction and gene regulation networks. These networks generally involve complex, nonlinear interactions of proteins, such as phosphorylation cascades (reviewed in [1]) and second messenger signaling systems [2], whose structures feature positive and negative feedback loops, feed-forward interactions, signal amplification, and cross-talk with other pathways [3]. Mathematical models of these interactions are therefore very insightful or even necessary avenues to analyze and understand the regulation of cell behavior, as the properties of these networks can exceed an intuitive understanding [4]–[6].

Notch is a signaling system required for numerous critical cell fate specification events during the development of the nervous system, hematopoietic system, eye, and skin [7]–[11]. The receptor for this pathway is the single pass transmembrane protein Notch that, when bound by its ligands Delta or Jagged, undergoes a series of cleavage events to release its intracellular domain (NICD) [9],[12]. This NICD then translocates into the nucleus and acts as a transcriptional upregulator of target genes, including members of the *hes* family, through its interaction with the transcription factor RBP-Jκ [13]. In mammals there are four different Notch proteins (Notch1-4) and 5 ligands (Delta 1, 3, and 4 and Jagged 1 and 2). For this study, we have focused primarily on the Notch1 signaling pathway.

In its role as a critical regulator of cell fate [7]–[11], Notch has been known to function via lateral inhibition and induction mechanisms to create fine-grained patterns in undifferentiated cells, a process required for proper boundary formation and differentiation of various tissues [14],[15]. It can also function as a binary cell fate switch, for example during differentiation of the epidermis [16] and endodermal epithelium of the gut [17], to promote differentiation of one cell type from precursor cells at the expense of another. Furthermore, in some cases continuous Notch activity is not required for cell fate specification. For example, transient Delta-Notch signaling has been shown to be sufficient to induce T-cell [18] and NK cell differentiation [19] from their respective precursor cells, and can induce an irreversible switch to gliogenesis in neural crest stem cells [20]. Notch signaling also occurs only transiently in many instances during the development of *Drosophila* [21], zebrafish [22],[23], and mice [24]. It was also recently shown that human embryonic stem cells (hESCs) require activation of Notch signaling to form the progeny of all three embryonic germ layers, and subsequent transient Notch signaling enhanced generation of hematopoietic cells from committed hESCs [25]. The mechanisms by which a short Notch signaling pulse can permanently switch cell fate are not elucidated.

The Notch system has also been shown to function as an oscillator. Specifically, the expression levels of members of the *hes* family, a group of downstream Notch target genes [26], have been shown to oscillate with a 2 hour periodicity in some systems during development, which for example aids in somitogenesis (i.e. the patterning of somites) [27]–[29]. Hes1 protein and mRNA concentrations have also been observed to oscillate with an approximate 2 hr time period upon serum starvation in various cultured cell lines including myoblasts, fibroblasts, and neuroblastoma cells [30]. Furthermore, oscillations in the Notch network have been proposed to be important in maintaining neural progenitor cells in an undifferentiated state [31]. Finally, there is evidence that such oscillations may also afford cells the opportunity to repeatedly test for the continued existence of a signal [32], thereby increasing cellular response sensitivity and flexibility by allowing the cell to integrate the results of many periodical evaluations of the signal before making an ultimate cell fate decision.

The Delta-Notch signaling system has been previously modeled to elucidate its role in fine-grained pattern formation through the action of lateral inhibition and induction [33]–[35]. Collier *et al.* developed a simple 2-parameter model that focuses on pattern formation due to feedback inhibition between adjacent cells via Delta-Notch signaling [33]. Other models build upon this simple model by adding more molecular detail at the intercellular level [34],[35]. In addition, several studies have focused on trying to understand the underlying mechanism of Notch system oscillations [32],[36], where a Hes1 negative feedback loop composed of Hes1 protein repressing *hes1* transcription, likely plays a central role [37]. Delays related to transcription and translation were also proposed to be important for the observed oscillations [38]. However, while several models have thus been proposed and have yielded important insights into this system [30], [36], [38]–[40], they have focused exclusively on Hes1 and not analyzed its interactions with other signaling proteins in the Notch system. Additionally, all these models focus on a particular aspect or mode of Notch signaling (e.g. lateral inhibition or oscillation) but do not yet address how complex, alternative behaviors could arise from the same network.

Here we mathematically model the Notch signaling system to analyze how the same network is capable of functioning as a cell fate switch or an oscillator in different biological contexts. This model, which includes the regulation of the *notch1-RBP-Jk-hes1* gene circuit, predicts that the Notch1-Hes1 system acts as a bistable switch in certain regions of parameter space, where Hes1 levels can change by 1–2 orders of magnitude as a function of the input Delta signal. In addition, it predicts that a transient pulse of a high level of Delta is capable of inducing high Hes1 expression levels for a duration that would be sufficient to induce a cell fate switch. Moreover, the model elucidates how the network can be ‘tuned’ to function in different regimes, either as an oscillator or a cell fate switch, by changing a key parameter. Finally, low numbers of reactants can lead to significant statistical fluctuations in molecule numbers and reaction rates, making cells intrinsically noisy biochemical reactors [41],[42]. Stochastic simulations of the Notch system, which enable the analysis of the effect of biological noise in the system arising due to stochastic variations in gene expression, reveal that for systems that respond quickly to Notch signaling, the network is able to dampen the effects of this biological noise and function in a manner similar to what is predicted by the deterministic model. In summary, the model enables analysis of the different behavioral responses of the Notch signaling network observed over a broad spectrum of signaling inputs and parameter values and can be further expanded to study Notch signaling in numerous contexts.

## Methods

**Figure 1:**
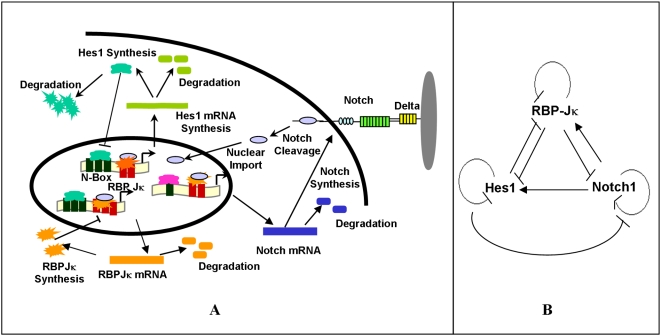
Schematic of the Notch1-RBP-Jκ-Hes1 signaling network. (A) Each arrow represents a term or event in the differential equation model including transcription, translation, mRNA and protein degradation, nuclear import, TF binding, receptor-ligand binding and receptor processing. (B) Schematic of the positive and negative feedback loops of the Notch1-RBP-Jk-Hes1 network. (-|) represents repression and (->) represents activation of target genes.

We developed a model of Notch signaling to investigate how this system can function as either an oscillator or as a simple binary switch capable of responding to steady state or transient inputs. Brief experimental work revealed that the *notch1* promoter is positively upregulated by its gene product and is downregulated by Hes1 (Text S1, Fig. S1). We thus examined the behavior of the *notch1*, *RBP-Jκ*, and *hes1* genes, which form a complex set of regulatory feedback loops (Fig. 1). A deterministic model composed of a system of differential equations was developed to analyze dynamic changes in the levels of the network constituents. However, since the concentrations of some of the species were low, stochastic simulations were also conducted to examine whether noise in the levels of the network components could significantly impact system behavior, as noise has the potential to undermine the fidelity of cell fate choices [41],[43].

### Deterministic Model Development

A set of differential equations was developed to track changes in the concentrations of various species in the nucleus and cytoplasm of a cell as a function of time following activation of Notch by its ligand. The cell is modeled as a 10 µm diameter sphere with a 5 µm diameter nucleus. Numerous processes were modeled as terms in the differential equation system, including transcription, translation, transport, degradation or - in the case of Notch - receptor cleavage (Fig. 1A, Text S1). As examples, the three equations tracking the Hes1 cytoplasmic mRNA, Hes1 cytoplasmic protein and Hes1 nuclear protein concentrations are given by:
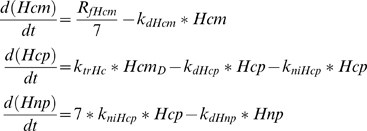
The rate of change (in units of 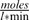) of the cytoplasmic mRNA concentration of Hes1 is given by the difference in the rates of it transcription and degradation. R_fHcm_ is the transcription rate of *hes1* mRNA in the nucleus. We assume instantaneous export of mRNA to the cytoplasm. A factor of 7 is included to take into account the dilution due to export to the cytoplasm (Text S1). k_dHcm_, k_dHcp_, and k_dHnp_ denote the degradation constants for the *hes1* mRNA (Hcm), cytoplasmic protein (Hcp), and nuclear protein (Hnp), respectively, which are assumed to undergo first order degradation kinetics. k_trHc_ denotes the translation constant (min^−1^) for conversion of cytoplasmic *hes1* mRNA into cytoplasmic protein. Transcriptional and translational delay times are incorporated into the model, as these are processes that inherently involve delays between initiation and the production of a molecule of mRNA or protein, as previously described [38],[44]. Thus, the translation of Hes1 protein is based on the delayed *hes1* mRNA concentration H_cmD_ (delayed by time TpHc, the average time for translation of Hes1), which is the concentration of mRNA present when the process of translation was initiated instead of the concentration at the present time. k_niHcp_ denotes the nuclear import rate in units of min^−1^. A dilution factor of 7 is again used to incorporate differences in nuclear and cytoplasmic volumes.

### The *hes1* Promoter

The transcription rates for *notch1*, *hes1*, and *RBP-Jκ* are based on the states of their respective promoters. Previous promoter analysis has been complemented with Genomatix Suite Gene2Promoter transcription factor (TF) binding site prediction software to identify potential TF binding sites in the promoters of the three genes in the model.

Takebayashi et al. [37] observed that *hes1* transcription is repressed by its own gene product through Hes1 protein binding to sites in the *hes1* promoter termed N-boxes. Through a series of binding and transcriptional activity assays, the study determined that Hes1 bound strongly to three N-boxes found upstream of the transcriptional start site and repressed transcription of the *hes1* mRNA up to 40-fold. Also, while the work concluded that there was a synergistic rather than an additive effect of the N-box binding dependent repression of gene expression, further mathematical analysis has indicated that there is no or very weak synergy among the different binding sites [45]. Several positive regulatory regions were also found in the *hes1* promoter, and it was also shown to have two adjacent RBP-Jκ binding sites [46],[47]. Thus, we have modeled the *hes1* promoter to have three equivalent N-boxes where the Hes1 protein can bind and repress transcription, as well as two equivalent RBP-Jκ sites. The presence of all other positive regulators of transcription is lumped into a constant basal rate of transcription.

### The *notch1* Promoter

As it has not been extensively investigated, the *notch1* promoter sequence was analyzed in the Gene2Promoter software. One putative Hes1 site (N-box) and two putative RBP-Jκ sites were found in the ∼1 kb *notch1* promoter analyzed. This may imply that *notch1* is both positively and negatively regulated by its own gene product. To test this, a transcriptional activity experiment using the dual luciferase assay system was conducted. The promoter of murine *notch1* [48] was used to drive expression of hRluc cDNA (Renilla luciferase). Co-transfection studies with plasmids expressing RBP-Jκ, NICD, Hes1, and dNHes1 (a dominant negative form of Hes1) indeed demonstrated that the *notch1* promoter is regulated negatively by Hes1 and RBP-Jκ in the absence of NICD but is positively regulated by NICD in the presence of RBP-Jκ (Text S1, Fig. S1). The *notch1* promoter was modeled with two RBP-Jκ sites and one N-box.

### The *RBP-Jκ* Promoter

A 418 bp sequence upstream of the *RBP-Jκ* gene as characterized by Amakawa et al. [49] was analyzed in the Gene2Promoter software for TF binding sites of interest. Three potential Hes1 binding sites and three potential RBP-Jκ sites were found. Thus, the *RBP-Jκ* gene also potentially undergoes autoregulation under Notch signaling, and a three N-box, three RBP-Jκ site model was utilized.

### Modeling the Transcription Term

As discussed above, all three promoters have one or more binding sites for both Hes1 (the N-box) and RBP-Jκ. It is assumed that Hes1 can bind and repress transcription of the corresponding promoter only in its homodimer form, and the dimerization reaction is assumed to be at steady state over timescales of protein transcription, translation and import, driven by mass action kinetics such that the concentration of the dimer is given by:
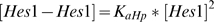
Where, K_aHp_ is the association equilibrium constant for the dimerization reaction. Similarly, the time scales of transcription factor binding to and dissociation from the promoter elements are also assumed to be much faster than those of gene transcription and protein synthesis, such that binding to the promoter is at pseudo steady state. In addition, it is assumed that NICD can bind only when an RBP-Jk protein is bound to its site on the promoter, and that this NICD binding converts RBP-Jκ from a transcriptional repressor to an activator [13].

The level of promoter activation (i.e. rate of mRNA synthesis) is modeled by an approach termed BEWARE [50],[51], in which the probabilities of a promoter being in any one of its many possible states are calculated based on the relative concentrations of the three transcription factors (Hes1, RBP-Jκ and NICD), and their respective DNA binding affinities, using equilibrium binding equations. The level of activation of the promoter is then given by: 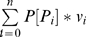, where, P[P_i_] is the probability of the promoter being in state i, and v_i_ is the activation rate of gene transcription associated to the promoter being in that state i. When the promoter is empty, the gene activation rate is assumed to be the basal transcription rate (V_b_) for that promoter. When a Hes1 dimer is bound to an N-box, the rate is reduced by a factor r_N_ that takes into account the repressive effect of the Hes1 transcription factor, and when RBP-Jκ is bound, the rate is reduced by a factor r_R_. Furthermore, when the promoter is in its maximally activated state with the NICD bound to the RBP-Jκ and no Hes1 dimers bound, the activation rate is assumed to be at its maximum and is given by (V_max_+V_b_). In the case of multiple RBP-Jκ binding sites, an additional factor t_c_ (<1) is used to account for states where not all RBP-Jκ sites bind NICD to represent the decrease from the maximum possible activation rate. For a detailed expression of transcription rates please refer to Supplemental Materials (Text S1).

Although explicit parameters have been included to account for cooperative binding for Hes1 dimers to multiple N-boxes and for RBP-Jκ binding (cooperativity factors C_n_, C_r_ and C_nr_ - please refer to Table 1 for model parameters), they have been set to 1 for these simulations, as recent work suggests there is very little if any cooperative effect in Hes1 binding to N-boxes [45]. Finally, it is assumed that each mRNA produces a fixed number of proteins, i.e. mRNA dynamics have been neglected [50].

**Table 1:** Parameter values used for the models.

Description	Symbol	Value	Source
Degradation constant of Hes1 protein (min^−1^)	kdHcp, kdHnp	0.0315	[30]
Degradation constant of Hes1 mRNA (min^−1^)	kdHcm	0.029	[30]
Degradation constant of RBP-Jk protein (min^−1^)	kdRcp	0.00231	[82]
Degradation constant of RBP-JK mRNA (min^−1^)	kdRcm	0.0075	[82]
Degradation constant of full-length Notch1 protein (min^−1^)	kdNp	0.017	[54], Text
Degradation constant of NICD protein (min^−1^)	kdNcp,kdNnp	0.0014 or 0.00385	[55], Text
Degradation of Notch mRNA (min^−1^)	kdNm	0.0058	[83]
Cooperativity factor for Hes1-DNA binding	Cn	1	Text
Cooperativity factor for RBP-Jk DNA binding	Cr	1	Text
Cooperativity factor for RBP-Jk Hes1 DNA binding	Cnr	1	Text
Rate of protein translation from Hes1 mRNA (min^−1^)	KtrHc	4.5	[45], Text
Rate of protein translation from RBP-Jk mRNA (min^−1^)	KtrRc	2.5	Text
Rate of protein translation from Notch1 mRNA (min^−1^)	KtrN	1	Text
RBP-Jk DNA association constant (M^−1^)	Kr	3.23×10^8^	[84]
Hes1 DNA association constant (M^−1^)	Kn	2×10^8^	Text
RBP-Jk NICD association constant (M^−1^)	Ka	1×10^8^	Text
Hes1 dimer association constant (M^−1^)	KaHp	1×10^9^	Text
Transcriptional time delay for Hes1 (min)	TmHc	10	[52], Text
Translational time delay for Hes1 (min)	TpHc	2.35	[52], Text
Transcriptional time delay for RBP-Jk (min)	TmRc	20	[52], Text
Translational time delay for RBP-Jk (min)	TpRc	4.3	[52], Text
Transcriptional time delay for Notch1 (min)	TmNc	70	[52], Text
Translational time delay for Notch1 (min)	TpNc	21	[52], Text
Basal transcriptional rate for Hes1 (M/min)	Vbh	1.14×10^−10^	[45], Text
Basal transcriptional rate for RBP-Jk (M/min)	Vbr	4.3×10^−11^	Text
Basal transcriptional rate for Notch1 (M/min)	Vbn	1.23×10^−11^	Text
Maximal transcriptional rate for Hes1 (M/min)	Vmaxh	5×10^−10^	[53], Text
Maximal transcriptional rate for RBP-Jk (M/min)	Vmaxr	2×10^−10^	Text
Maximal transcriptional rate for Notch1 (M/min)	Vmaxn	5.5×10^−11^	Text
Nuclear import rate of Hes1 protein (min^−1^)	kniHcp	0.1	[85], Text
Nuclear import rate of RBP-Jk protein (min^−1^)	kniRcp	0.1	[85], Text
Nuclear import rate of NICD protein (min^−1^)	kniNcp	0.1	[85], Text
NICD generation constant upon Delta binding (M^−1^ min^−1^)	KfNcp	7.6×10^7^	Text
Repression constant of Hes1 bound to N-box	rNbox	0.3	[30], Text
Repression constant of RBP-Jk alone bound to promoter	rR	0.2	Text

### Parameter Determination

Experimentally determined values for half-lives of proteins and mRNA, association and dissociation constants of proteins to their respective DNA binding sites, dimerization constants, and protein translation and transcription rates have been used when possible (Table 1). These values are often not available for the exact species of interest; however, the best available estimates based on similar protein classes are used wherever applicable as the starting point. The time delays for transcription and translation for each of the three genes are calculated as previously described [52] and are detailed in the Supplemental Materials (Text S1). 4.5 transcripts per minute [45] and 20 transcripts per minute [53] were used as initial estimates for *hes1* basal and maximum transcription rates respectively. The transcription rates for *RBP-Jκ* and *notch1* were then determined from these estimates and the estimates of their minimum transcription times (Text S1).

The degradation rates for the Hes1 protein and mRNA were determined experimentally by Hirata et al. in fibroblasts [30]. They observed similar values in other cultured cell types including myoblasts, neuroblastomas, and teratocarcinomas. Pulse chase experiments of Logeat et al. [54] were used to assess the degradation rates for the full-length Notch1 protein, and an estimate of Notch1 protein half-life of ∼40 minutes was derived.

GSK3β has been shown to affect the stability of NICD [55]. Although there are conflicting results as to whether GSK3β helps to stabilize [55] or destabilize the cleaved NICD [56], our experimental results show that GSK3β is essential for the NICD regulation of neural stem cell differentiation into astrocytes (Agrawal, Ngai, and Schaffer, manuscript in preparation). Furthermore, we show that Notch1 signaling upregulates the expression of GSK3β in these cells. Thus, the effect of GSK3β is incorporated into the model by increasing the half-life of NICD from 3 to 8 hrs [55] above a threshold concentration of Hes1 (which is assumed to directly or indirectly regulate the expression of GSK3β). This increased NICD half-life does not however change the qualitative behavior of the Hes1 switch (Fig. S3A).

The repression constant of Hes1 dimer bound to an N-box (r_Nbox_) is estimated from the results of Takebayashi et al. [37] that show that in the presence of three N-boxes, transcription is repressed by ∼40 fold. This yields a repression value of ∼0.3 per N-box (Please refer to Supplemental Materials (Text S1) for details). Since there are no reliable estimates of the NICD generation constant upon Delta binding (k_fNcp_), a lumped parameter of this constant with the Delta concentration is used to report the strength of the Delta signal (k_fNcp_*Delp). The initial parameters for which the experimentally determined values are not accurately available were later subjected to sensitivity analysis (See results).

### Computational Methods and Initial Conditions

The differential equations described in the model were solved (with parameter values given in Table 1) using Berkeley Madonna 8.3.11 software (www.berkeleymadonna.com) with the Runge Kutta 4 module at a step size of 1 min. To arrive at realistic initial conditions for the model, the initial concentrations of all species were set to 0 with zero Delta signal, and the simulations were run until the various species attained steady state concentration levels. These steady state values (listed in Table 2) were then used as the initial conditions for subsequent simulations. For the various experiments, the system was run for 750 minutes without stimulation with the Delta ligand to attain a basal steady state, and the Delta concentration was then increased to different levels to initiate Notch1 signaling. Simulations were run either with a constant Delta signal throughout or with varying duration pulses of the Delta signal. The system was simulated for a duration of 5,000–10,000 minutes (∼3.5–7 days), as neural progenitor stem cells have been previously shown to undergo differentiation upon Notch activation in 3–5 days ([57]). Longer simulations up to 50,000 minutes were conducted when required to confirm Hes1 had reached steady state levels.

**Table 2:** Initial conditions for deterministic and stochastic models.

Species	Deterministic Model (mol/l)	Stochastic Model (# of molecules/cell)
Hcm (Hes1 mRNA)	4.34 * 10^−12^	1
Hcp (cytoplasmic Hes1 protein)	1.48 * 10^−10^	41
Hnp (nuclear Hes1 protein)	3.30 * 10^−9^	130
Rcm (RBP-Jκ mRNA)	1.44 * 10^−12^	1
Rcp (cytoplasmic RBP-Jκ protein)	3.53 * 10^−11^	10
Rnp (nuclear RBP-Jκ protein)	1.07* 10^−8^	422
Nm (Notch1 mRNA)	1.66 * 10^−11^	5
Np (Notch1 protein)	9.74 * 10^−10^	269
Ncp (cytoplasmic NICD)	0	0
Nnp (nuclear NICD)	0	0

### Stochastic Model Development

Since the levels of several protein species in the deterministic model simulations were very low (Table 2), at the level of tens of molecules per cell, assumptions of mass action kinetics and pseudo steady state may not hold true, and stochastic effects may play an important role in the dynamics of the signaling network [41],[58]. To analyze whether noise in protein and mRNA concentrations would impact the dynamics of the system, a stochastic simulation of the model using the Gillespie algorithm [43] was implemented in C++ (code available upon request). To relax the assumptions of mass action kinetics and pseudo steady state, we explicitly simulated every reaction step, making a total of 299 reactions. For example, every interaction between a transcription factor and a promoter was modeled as a discrete reaction in the simulation. The τ-leap method [59] was also incorporated into the algorithm to accelerate the stochastic simulations and increase their efficiency.

## Results

### The Notch1-Hes1 Network as a Bistable Switch

**Figure 2:**
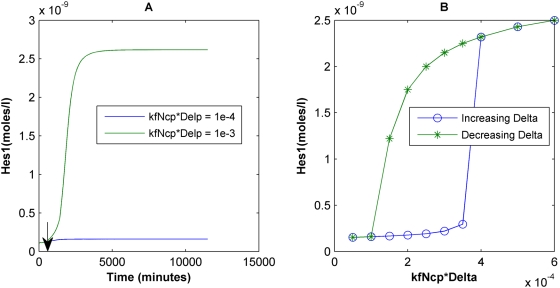
Bistability in Notch signaling. (A) Deterministic Hes1 trajectories as a function of time for two different strengths of Delta signals given as a product of the Delta concentration and the rate constant of formation of NICD upon Delta-Notch binding (kfNcp*Delp = kDelp). These deterministic simulations were initiated using steady state values of the system under no Delta signal and at t = 750 minutes (indicated by vertical arrow) the input Delta signal was applied. (B) Hysteresis in the Notch1-Hes1 network, where Hes1 concentration can attain two possible steady states for an intermediate range of Delta inputs. The point of switching depends on whether the Delta signal is increasing or decreasing.

The response of the Notch1-RBP-Jκ-Hes1 system to a step change in an input Delta signal was analyzed. Simulations were initiated using the steady state levels of the different species in the absence of any external Delta (also listed in Table 2), and at t = 750 minutes a Delta signal was applied. Fig. 2A demonstrates that when a low input Delta stimulus is applied, the Hes1 concentration settles to a correspondingly low steady state value. However, when the input Delta signal was increased (10-fold), Hes1 shows a rapid increase to a new, 20-fold higher steady state value. Further steady state analysis at a range of input Delta levels and initial conditions reveals that the system exhibits bistability. At low levels of Delta signal, basal levels of Hes1 are maintained in the cell (“OFF” state), but as the Delta signal strength is increased beyond a threshold level, it stimulates the production of Hes1, which is then maintained at high levels (“ON” state) through the concerted regulation of the Notch1-RBP-Jκ-Hes1 network (Fig. 2B). Bistability – which has previously been proposed as an advantageous mechanism to mediate an unambiguous cell fate switch, including in stem cells [51],[60] – is evident within an intermediate range of Delta signal values (Fig. 2B).

### Network Sensitivity to Biological Noise

**Figure 3:**
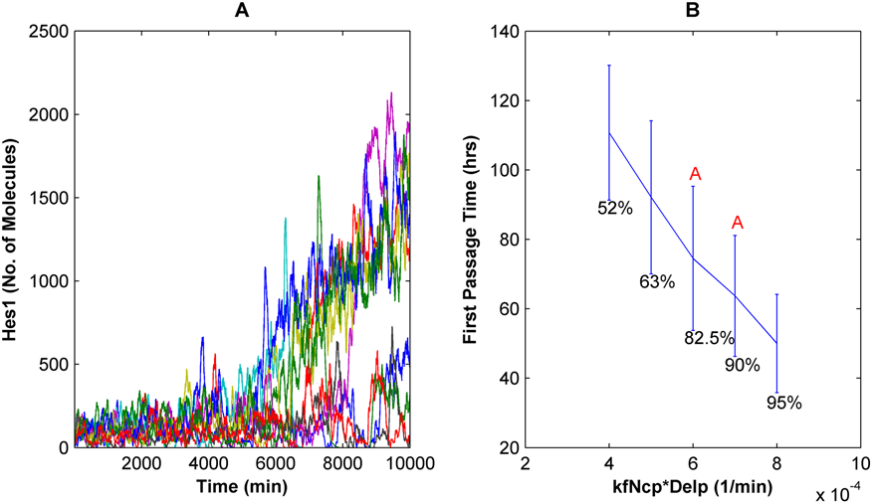
Stochastic simulations demonstrate spontaneous “OFF” to “ON” transitions. (A) Representative stochastic Hes1 trajectories as a function of time after application of a constant Delta stimulus at 750 min at levels just below “ON” levels predicted by the deterministic model. Some Hes1 trajectories remain at low levels (“OFF” state) while others randomly switch state to higher levels (“ON” state). (B) First passage time (FPT) of stochastic trajectories for passage from “OFF” to “ON” state as a function of Delta signal strength in the bistable region. The mean and standard deviation of 40–60 runs in each case are plotted. The percentage of trajectories that switched to “ON” state under the given Delta signal is indicated below each data point. All points except those connected by the same letter (A) are statistically distinct (p<0.01, 2-tail t-test).

The initial numbers of some protein and mRNA species in the system were in the range of tens of molecules per cell (Table 2), such that stochastic fluctuations in individual species may impact the dynamics of the network. In particular, intracellular noise inherent in systems with small numbers of molecules and/or slow biochemical reactions can randomize or undermine the “accuracy” of cell fate choices [41],[58]. To analyze such behavior, stochastic simulations based on the Gillespie algorithm [43], distinct from the deterministic model, were developed. Steady state analysis shows that at low, constant Delta signals, the Hes1 levels fluctuate about a low mean value corresponding to the “OFF” state, as expected (data not shown). However, if the Delta signal is increased to a level just below the concentration at which the deterministic model would predict a switch in state (Fig. 2B), stochastic simulations reveal that noise in the network can induce some trajectories to spontaneously switch states (Fig. 3A). Analogous to results previously observed in other systems [51],[61],[62], noise thus undermines the bistable switch and induces spontaneous flipping between states. Analysis of the time it takes the system to initially pass from the lower to the upper state reveals that as the strength of the input signal is increased, this average first passage time (FPT) decreases, and the percentage of trajectories that change state increases (Fig. 3B). However, this “uncertainty” occurs within a narrow range of intermediate Delta signal levels, and if this intermediate window is avoided, the system effectively behaves deterministically.

**Figure 4:**
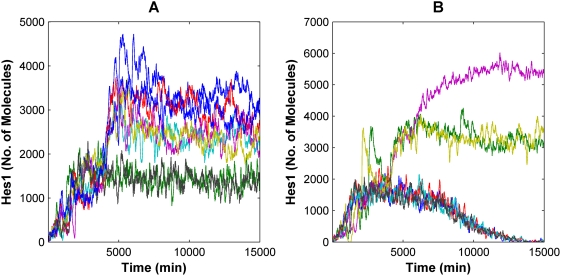
The Notch system exhibits bistability under stochastic simulations. (A) Hes1 stochastic trajectories are shown during high Delta levels for 4000 minutes, following which the Delta signal is brought down to levels that failed to switch the state to “ON” when provided for a prolonged duration (kDelp = 4×10^−4^) in the deterministic model. All the trajectories remain in the “ON” state – corresponding to the region of bistability seen in the deterministic simulations. (B) Hes1 stochastic trajectories are shown after application of a high Delta signal for 4000 minutes, after which the Delta signal is brought down to 0. Some trajectories persist in the “ON” state.

In addition, “ON” to “OFF” transitions were simulated by first stimulating with a high Delta signal for 4000 minutes to induce high Hes1 expression levels. When Delta was then reduced to levels that were in the predicted bistable region based on the deterministic model, the system maintained high expression levels of Hes1 (Fig. 4A), as anticipated from the deterministic results (Fig. 2B). Contrary to what was expected based on the deterministic model, however, when the Delta signal was instead reduced to zero, some trajectories remained in the high Hes1 expression (“ON”) state (Fig. 4B). This indicates the role of stochastics in potentiating high Hes1 expression levels even in the absence of continued signal.

### Response of the System to Transient Delta Activation

It has been shown for neural crest stem cells [20] that a transient Notch signal is sufficient to induce cell differentiation. Also, there are numerous situations where transient Notch-Delta signaling determines the fates of immature cells, both in tissue culture [18],[19] and during organismal development [21]–[24]. Under continuous Delta stimulation, the system can attain high steady-state Hes1 expression levels, thus acting as a switch, but we next wanted to examine whether transient Delta activation was also capable of eliciting high Hes1 expression. We thus examined the dynamic response of the system to transient activation of the Notch1 pathway upon variation in the strength and duration of an applied Delta signal.

**Figure 5:**
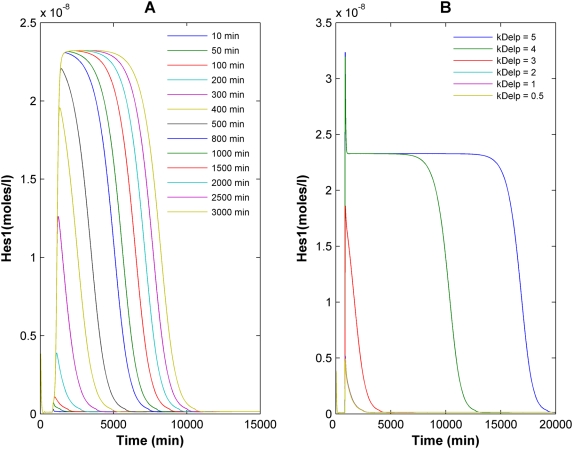
Both input Delta signal strength and duration affect the output Hes1 expression levels. (A) Effect of Delta signal duration on the Hes1 expression levels: a transient Delta signal of kfNcp*Delp = 5×10^−3^ was provided in the deterministic model for varying amounts of time ranging from 10 minutes to 3000 minutes, and the resulting Hes1 trajectories were simulated up to 15000 minutes. (B) The effect of Delta signal strength on Hes1 expression: a transient Delta signal of varying strengths (expressed as kDelp = kfNcp*Delp (min^−1^)) was provided in the deterministic model for 100 minutes, and the resulting Hes1 were simulated up to 20000 minutes.

When the system is stimulated for a short duration (10 minutes) with a moderate strength Delta signal, the deterministic model predicts a transient peak in the Hes1 expression that eventually decays to its low steady state value (Fig. 5A). However, the peak expression of Hes1 continually increases with increasing input signal duration up to ∼800 minutes, beyond which the maximum expression levels of Hes1 attained remain the same but the duration of prolonged high expression levels progressively increases (Fig. 5A). Similarly, as the input Delta signal strength is increased for a constant pulse duration, the peak Hes1 concentrations attained also increase up to a maximum value, after which a further increase in the signal strength only increases the duration of high Hes1 levels (Fig. 5B). The cell is thus able to attain high Hes1 expression either under prolonged low intensity Delta signaling or a short burst of high intensity Delta signaling.

### Stochastic Effects on Transient Delta Signaling

**Figure 6:**
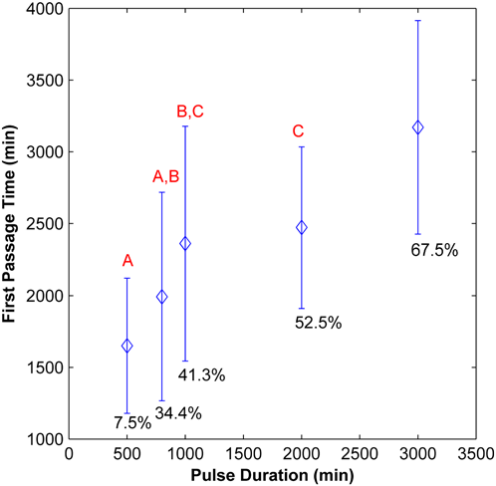
First passage time (FPT) for passage from “OFF” to “ON” state as a function of Delta signal duration in stochastic simulations. The mean and standard deviation of >20 runs in each case are plotted. The percentage of trajectories that switched to the “ON” state under the given Delta signal is indicated below each data point. All points except those connected by the same letter (A,B,C) are statistically distinct (p<0.01, 2-tail t-test).

We also examined the effect of stochastics on transient activation of the network. Simulations were run using the parameter values as in the deterministic model for various Delta pulse durations ranging from 10 minutes to 3000 minutes and >40 trajectories per input duration value were analyzed. For Delta pulse durations of less than 500 minutes, the stochastic simulations followed the prediction of the deterministic model (data not shown). However, for a 500-minute Delta pulse, even though the deterministic model predicts a transient Hes1 peak that does not attain the maximum possible expression level, a small percentage of the stochastic trajectories in fact did switch to the “ON” state (corresponding to high Hes1 expression levels) (data not shown). Also, as the duration of the Delta pulse is increased, the percentage of trajectories that remain in the “ON” state for the simulated 15,000 minutes progressively increases even though the deterministic model predicts that the system would revert back to the “OFF” state within that time. Furthermore, the average first passage time (FPT) of the trajectories that do switch state increases as the Delta pulse duration increases (Fig. 6). It is likely that for shorter Delta pulse durations, if the system is to undergo the spontaneous “OFF” to “ON” transition, it does so early, soon after the application of the Delta signal. However, in the case of longer duration input signals, the continued presence of the signal allows trajectories to switch state even much later in the simulation, resulting in an apparently longer first passage time. Collectively, these results imply that even for very short signal pulse, a small fraction of a population of cells receiving a pulse of Delta signal could switch their state due to stochastic effects.

### Bifurcation Analysis

**Figure 7:**
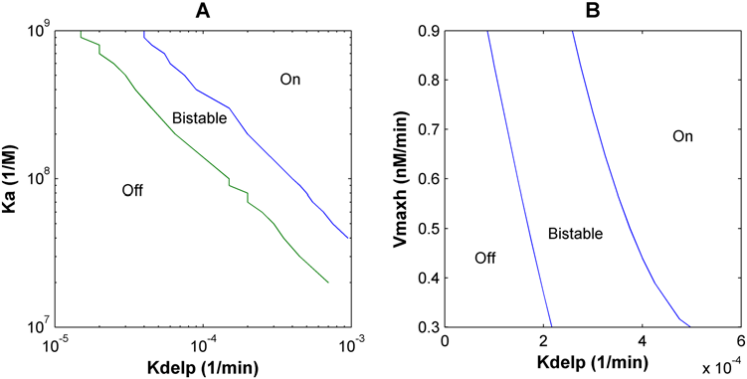
Bifurcation Analysis. (A) Bifurcation analysis of how the switching points vary with the equilibrium binding constant (K_a_) of NICD to RBP-Jk. Stronger interaction between NICD and RBP-Jk lowers the threshold of Delta signal required to turn the system ON. (B) Bifurcation analysis of how the switching points vary with the maximal transcription rate of Hes1 (V_maxh_). A higher maximal transcription rate, indicating a stronger Hes1 promoter, also slightly shifts the region of bistability towards lower Delta signal strengths.

A number of parameters in the model have not been directly experimentally measured and were estimated from data available for similar protein classes in different contexts, and we thus performed sensitivity analysis for all such parameters by varying them individually through a broad range of values in the deterministic model (Table 3, Fig. S2). Although in most cases the qualitative behavior of the system remained unchanged, the system did exhibit considerable sensitivity to specific parameters, which were then subjected to further analysis. These include: the half-life of NICD, the equilibrium binding constant of NICD with RBP-Jκ (K_a_), the maximal transcription rates (V_max_), and the repression constant of Hes1 (r_Nbox_). NICD has a long half-life of a few hours under normal physiological conditions [55]. However, our model indicates that if the NICD half-life is drastically reduced, the system fails to function as a switch and cannot express high levels of Hes1 (Fig. S3). In addition, the equilibrium binding constant (K_a_) of NICD to RBP-Jκ in the model is 10^8^ M^−1^, but as K_a_ increases – denoting stronger interactions of NICD with the promoter – bifurcation analysis demonstrates that the OFF-ON transition occurs at accordingly lower values of the Delta signal (k_Delp_) (Fig. 7A). Similarly, increasing the maximal transcription rate of Hes1 (V_maxh_) to indicate a stronger promoter shifts the OFF-ON transitions to lower Delta signal strengths (Fig. 7B).

**Table 3:** Summary of results of sensitivity analysis documenting the effect of increasing parameter values on the threshold of Delta signal strength required to switch the system state from OFF to ON.

Parameter	Range of variation	Effect on threshold Kdelp (Delta signal strength) with increasing parameter value	Results
Ka	10^7^–10^9^ (M^−1^)	Decrease over 2 orders of magnitude; no qualitative effect	Fig. 7A
Vmaxh	0.3–0.9 nM/min	Slight decrease; no qualitative effect	Fig. 7B
rNbox	0–1	Drastic qualitative change in behavior of the system	Fig. 8
Kn	10^7^–10^9^ (M^−1^)	Slight increase; no qualitative effect	Fig. S2A
KaHp	10^8^–10^10^ (M^−1^)	Slight increase; no qualitative effect	Fig. S2B
rR	0–0.5	Slight decrease; no qualitative effect	Fig. S2C
kdNcp, kdNnp	0.001–0.04	Increase over 2 orders of magnitude	Fig. S3

The system exhibits a shift in the region of bistability, thus changing the sensitivity of the system to the Delta signal, but the qualitative nature of the gene network in most cases remains the same for a broad range of the parameter values.

### The Degree of Repression by Hes1 Determines the Qualitative Nature of the Cellular Response to Delta Stimuli

**Figure 8:**
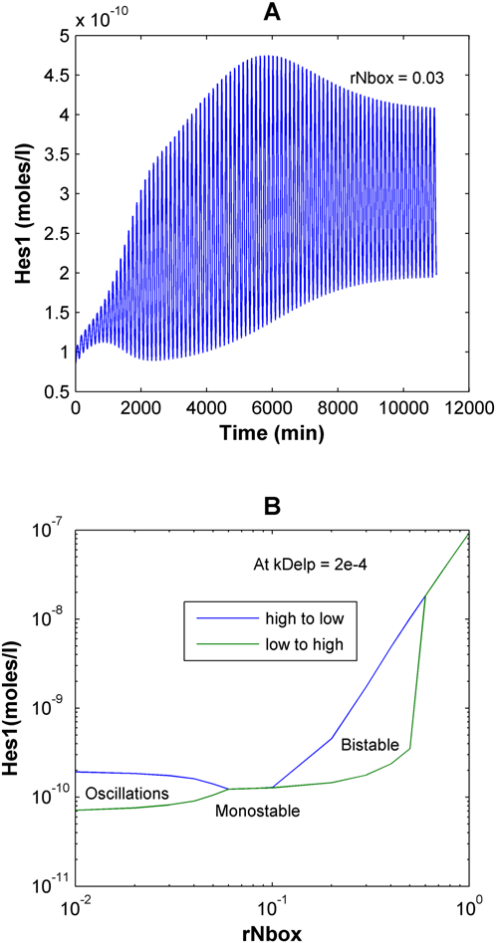
Effect of the repression constant of Hes1 (r_Nbox_) on the Notch signaling network. (A) At lower values or r_Nbox_ (higher repression constants for Hes1), the network predicts oscillations in Hes1 levels. As the value of r_Nbox_ is decreased to 0.03 and lower, the system exhibits stable oscillations. (B) At a fixed Delta signal strength of kDelp = 2×10^−4^, as the r_Nbox_ is progressively decreased, the response of Hes1 transitions from behaving as a bistable switch to a brief region of monostability to an oscillator.

Interestingly, the response of the deterministic model was most sensitive to the extent to which Hes1 binding reduced or repressed expression of target genes (r_Nbox_). As the Hes1 repression constant (r_Nbox_) is progressively decreased (or the repressive strength of Hes1 progressively increased) from 0.3 to 0.1, the final steady state concentrations of Hes1 progressively decrease for a given level of Delta signaling (Fig. S4), but the system continues to exhibit bistability. Intriguingly, as the value of r_Nbox_ is further decreased below 0.1, there is a dramatic qualitative change in the response of the system. Specifically, the system undergoes a bifurcation or transition from bistable to monostable behavior and at such high repressive strengths is unable to attain high steady state Hes1 expression levels. Finally at very low values of r_Nbox_ (<0.03), it once again undergoes a transition to a stable oscillatory response where the Hes1 levels in the cell oscillate about a low mean steady state value (Fig. 8A). A phase plot of the response of the system with variable r_Nbox_ (Fig. 8B) demonstrates how the same gene network can transition from behaving as a bistable switch to being an oscillator. The model thus elucidates the versatility of the system, where tuning of a single key parameter can convert its behavior from a switch to a clock. Previous *hes1* models showing sustained oscillations have focused exclusively on the low r_Nbox_ region (i.e. r_Nbox_ = 0) of such a phase plot [30],[36],[38],[39].

## Discussion

The Notch signaling system is an evolutionarily conserved network that functions in multiple organs to orchestrate cell fate specification [63]–[65] in a context dependent manner. In some cases, it can function as a binary cell fate switch at the individual cell level [16],[17], whereas in other situations cell-cell contact dependent Notch signaling can result in pattern formation in an array of cells [14],[15], and in yet other contexts it can function as a biological clock to govern pattern formation and differentiation during somitogenesis [27]–[29]. Although several additional components such as Fringe, Numb, and Presenilin can feed into and modulate the Notch signaling cascade, the core of the signaling pathway is relatively simple, where Notch acts as a membrane bound transcription factor that is activated by ligand binding and induces transcription of target *hes* genes via its interaction with the RBP-J*k* transcription factor [10]. However, the system can exhibit complex inter-regulation of its components. A better understanding of the functioning and regulation of this signaling system – and in particular how it exhibits diverse behaviors in different contexts – is valuable from a basic biology standpoint, in understanding how misregulation of the Notch signaling pathway can underlie disease, and from regenerative medicine viewpoint in therapeutic applications of stem cells.

Mathematical modeling can provide valuable insights into the behavior of this gene regulatory circuit. Previous models have focused either on the level of cell-cell interactions to simulate the levels of Notch and Delta within adjacent cells and thereby analyze pattern formation based on levels of Delta and Notch levels in an array of cells [33]–[35], or on the autoregulation of the *hes* genes in isolation to examine the oscillatory behavior of the gene circuit [30],[36],[39],[40],[44],[45],[66],[67]. Here we have developed an integrative model that takes into account the intracellular signaling network downstream of Notch activation through its ligand Delta, leading to the activation of the *hes1* gene via interaction with RBP-Jκ. These three genes potentially regulate the transcription of one another (Text S1, Fig S1) [37],[46],[47], forming a network of positive and negative feedback loops (Fig. 1B). Our model begins to elucidate how a cell can potentially tune key system parameters in the resulting Notch1-Hes1 gene circuit to elicit diverse responses.

The behavior of the system was most sensitive to the repression constant of Hes1, r_Nbox_. The degree of Hes1 repression of a transcriptional target can be modulated by the presence of co-factors. For example, whereas Groucho can act as a transcriptional co-repressor for Hes1, Runx2 can act as a negative regulator of the repressive activity of Hes1 by interfering with the interaction of Hes1 with the TLE corepressors [68]. The repressive activity of Hes1 can also be further potentiated by its interaction with the winged-helix protein brain factor 1 [69]. Therefore, because different cells can express these factors to different extents, which can thereby modulate the value of r_Nbox_, the same gene circuit can be tuned to transduce an input Delta signal into qualitatively different responses – oscillation vs. switching.

The model predicts that for low repressive strengths of Hes1 (0.1<r_Nbox_<0.3), the Hes1 expression level functions as a bistable switch in response to varying the strength of the Delta signal, thereby providing an unambiguous fate switch that is insensitive to the presence of small fluctuations in input signal (Fig. 2). Hysteresis has been previously observed experimentally in other biological systems including the JNK signaling cascade [70],[71] and the Cdc2 cell cycle regulation [72]. Parameters such as K_a_ (the association binding constant of NICD to RBP-Jκ) and V_max_ (the maximal transcription rates) can shift the region of bistability, thus changing the sensitivity of the system to the Delta signal, but the qualitative nature of the gene network remains the same for a broad range of these parameter values. Positive feedback loops with nonlinearity can yield bistability [51], and both Notch1 autoregulation and NICD-mediated conversion of RBP-Jκ into a transcriptional activator that in turn upregulates Notch1 expression constitute positive feedback loops that can drive this behavior.

Since the numbers of some protein and mRNA species in the model were low (Table 2), we developed a stochastic model to examine the effect of biological noise and cell-to-cell variability on the bistable response of the system to Delta signaling. Spontaneous OFF to ON switching of states was observed even in regions not predicted by the deterministic model. For example, as the Delta values are increased through the bistable range, the percentage of trajectories switching to the ON state increases, and the average FPT for these trajectories decreases (Fig. 3B). These results are consistent with observations in other bistable systems [73], and computationally in other signaling systems [51], where noise has been shown to cause spontaneous switching of states. However, since the timescale of a system's downstream response to the Notch network's state varies from a few hours (for example during somitogenesis) [74] to a few days (for example during stem cell differentiation) ([57]), the impact of stochastic noise on the fate switch will also be different in different contexts. Thus, for very low Delta signals, the average FPT is sufficiently high (>110 hrs) such that the cell remains in the OFF state for prolonged periods of time and would be non-responsive to Delta signaling over timescales of a few hours, whereas in the case of a population of cells experiencing Notch signaling over a period of 4–6 days, spontaneous switching could undermine the genetic switch and cause some cells to change fate at these low Delta input signals.

While the system can behave as a switch in a particular range of parameters at steady state, there are also many situations in which Notch signaling is transient, yet is sufficient to induce a switch in cell fate [18]–[24]. To simulate this, the model behavior was analyzed under transient Delta activation. The network response to a transient Delta stimulus was a strong function of both the signal intensity and duration, and either a high intensity signal for a short duration or a low intensity signal for a prolonged duration was capable of inducing transient increase in Hes1 expression levels for up to 2.5 days after withdrawal of the signal (Fig. 5), a time sufficient to initiate a biological response [57].

This prolonged expression of Hes1 upon transient Delta activation is due to the long half-life of NICD [55]. The bistable switch is thus sensitive to the degradation constant of NICD. If the NICD half-life were for example drastically reduced, the model would predict that the system would fail to express high levels of Hes1 regardless of Delta levels (Fig. S3). Hes1 is a repressive transcription factor that in some systems plays a crucial role in suppressing the activation of oncogenes. For example, in breast cancer cells, Hes1 can inhibit both estrogen- and heregulin-beta1-stimulated growth via downregulation of E2F-1 expression [75]. Thus, a malfunction in the Notch system, such as a reduction in NICD half-life, could contribute to cell transformation. Indeed, aberrant Notch signaling is implicated in many cancers (reviewed in [76]). For example, integrin-linked kinase (ILK), which is either activated or overexpressed in many types of cancers including breast cancer [77], can remarkably reduce the protein stability of Notch1 and thus decrease its half-life drastically [78]. Interestingly, high ILK and low NICD levels are detected in basal cell carcinoma and melanoma patients [78].

By increasing the repressive strength of the Hes1 dimer by 10-fold (r_Nbox_<0.03), the cell can transition from being a bistable system, to a brief region of monostability, and finally to an oscillator (Fig. 8B). Oscillations occur with a time period of approximately 2 hrs, similar to what Hirata et al. observed in cell culture [30]. This value also compares well with the various models that have been developed (for the Hes system in isolation) to explain oscillations in the *hes* family of genes and their homologues. These models assume complete repression in the presence of even a single Hes homodimer bound to the promoter region [36],[39],[45],[66]. This corresponds to an r_Nbox_ value of 0, in which case there would be no difference between the repressive strength of promoters with 1, 2 or 3 N-boxes. From the experimental observations of Takebayashi et al. [37], where the repressive strength of the promoter did in fact increase with the number of N-boxes, the estimated value of r_Nbox_ is 0.3. However, during somitogenesis, the factors expressed in the presomitic mesoderm (PSM) may enhance the repression due to Hes1 such that the value of r_Nbox_ is very low.

This current model represents the Notch signaling network core in a single cell, and it can readily be extended to a field of cells to analyze the role of Notch in patterning tissue formation [60]. In addition, there are numerous cell-specific mechanisms and factors that feed into this important signaling core [79]–[81]. Additional molecular species can be added to this model framework, or the parameter values of the current model can readily be modulated for example to simulate changes in DNA binding affinities, repressive constants, or the protein and mRNA stabilities as a function of cell-specific factors. This simple but versatile model can therefore be expanded by incorporation of additional molecular mechanism, specific to particular cell types, to make predictions on the role of Notch signaling in diverse cells and tissues.

In summary, we have theoretically and computationally analyzed the Notch1-RBP-Jκ-Hes1 signaling network, which is responsible for cell fate specification in numerous contexts. Our results indicate that the network, consisting of both positive and negative feedback mechanisms, can be tuned to function either as a bistable cell fate switch or an oscillator based on relatively small changes in a key parameter value. Furthermore, the duration and strength of the Delta signal regulate either the peak or the final steady state levels of Hes1 attained. Therefore, cells can readily tune the Notch system to regulate a variety downstream cell fates and functions.

## Supporting Information

Text S1 Supplemental Materials
(0.09 MB DOC)

Figure S1 Transcriptional analysis of the Notch1 promoter. Relative fold changes in the activity of the Notch1 promoter in the presence of exogenous Hes1 (H), dNHes1 (dN), RBP-Jκ(R) and NICD (N) are shown. Relative amounts of plasmids used in each case encoding the respective cDNA are indicated. For example H0.1dN1R0.5N0.5 indicates Hes1 = 0.1 µg, dNHes1 = 1 µg, RBP-Jk = 0.5 µg and NICD = 0.5 µg in a total of 4 µg transfection.
(2.52 MB TIF)

Figure S2 Bifurcation Analysis. (A) Bifurcation analysis of how the switching points vary with the Hes1 DNA association constant (Kn). Varying the association constant over two orders of magnitude causes a slight shift in the strength of the Delta signal required to switch the system state. Thus, stronger DNA association of Hes1 (higher values of Kn) increases the threshold values of Delta signal strength (Kdelp) required to turn the system ON. (B) Bifurcation analysis of how the switching points vary with the Hes1 dimerization constant (KaHp). Varying the dimerization constant over two orders of magnitude causes a slight increase in the strength of the Delta signal required to switch the system state to ON. (C) Bifurcation analysis of how the switching points vary with the repression constant of RBP-Jκ (rR). Increasing the repression constant from 0 to 0.5 (corresponding to a decrease in the RBP-Jκ repressive strength), has very little effect on the threshold of Delta signaling strength required to turn the system ON.
(0.82 MB TIF)

Figure S3 Effect of half-life of NICD on the Hes1 switch. (A) Changing the half life from 3 hrs (kdNcp = 0.00385) to 8 hrs (0.0014) due to the effect of GSK3β causes a slight increase in the steady state Hes1 concentration in response to a Delta signal of kDelp = 5×10^−4^, but no qualitative change in the switch. Increasing the degradation constant by 10-fold (kdNcp = 0.0014 to 0.014) however, causes complete suppression of the switch. (B) Analysis of how the threshold value of Delta signal required to switch the system from OFF to ON increases with the increasing degradation constant of NICD (decreasing NICD half life).
(0.52 MB TIF)

Figure S4 Effect of repression through Hes1 (r_Nbox_) on the high steady state values of Hes1 expression.Decreasing r_Nbox_ progressively decreases the steady state concentrations of Hes1 in the r_Nbox_ range of 0.3 to 0.1.
(0.42 MB TIF)
