# Supplementary material for: Computational Models of the Notch Network Elucidate Mechanisms of Context-dependent Signaling
Source: PLoS Comput Biol. 2009 May 22;5(5):e1000390. doi: 10.1371/journal.pcbi.1000390 (PMC2680760; doi:10.1371/journal.pcbi.1000390)
Supplement: Text S1 — Supplemental Materials (0.09 MB DOC) [file pcbi.1000390.s001.doc]

**Computational Models of the Notch Network Elucidate Mechanisms of Context-dependent Signaling**

**SUPPLEMENTAL MATERIALS**

**Cell culture, plasmids and dual luciferase assay**

The *notch1* promoter was analyzed for transcriptional activity by a luciferase assay. HEK 293T cells were cultured in Iscove’s Modified Dulbecco’s Medium (IMDM) (Hyclone) supplemented with 10% fetal bovine serum (FBS) (Invitrogen) and 1% penicillin-streptomycin (Gibco). The luciferase assay was performed using the dual luciferase assay kit from Promega following manufacturer’s protocols. The *notch1* promoter sequence from the Notch-XHO-GFP construct [1] (a generous gift from Dr. Wei-Qiang Gao) was used to drive the expression of the hRluc (Renilla luciferase) gene. 1 μg of the Notch1-hRluc plasmid was co-transfected into the HEK 293T cells with 0.5 μg CLPIT luc plasmid (a plasmid constitutively expressing the luciferase gene) and varying amounts of Hes1, dNHes1, RBP-Jκ, and NICD expressing plasmids using the calcium phosphate method.

The RBP-Jκ expressing plasmid was a generous gift from Dr. Diane Hayward [2]. The CPLIT NICD vector was created by excising out the NICD fragment from the PCS2-ICV-6MT plasmid (a generous gift from Dr. R. Kopan) [3,4] using *Hind* III and *SnaB* I restriction enzymes and ligating to *Hind* III and *Hinc* II of PBS SK SP. The resulting PBS NICD was further digested with *Sfi* I/*Pme* I restriction enzymes and ligated into the MLV retroviral CLPIT (CMV LTR Puro IRES TetO) vector also digested with *Sfi* I/*Pme* I. The CLPIT Hes1 vector was created by first excising out the *hes1* cDNA from the PCI-Hes1 plasmid (a kind gift from Dr. R. Kageyama) using the *EcoR* I restriction enzyme and cloning it into the PBS SK SP vector using the *EcoR* I site, followed by digestion of the PBS Hes1 plasmid with *Sfi* I/*Pme* I restriction enzymes and ligation into the CLPIT vector also cut with *Sfi* I/*Pme* I. The dNHes1 plasmid was a kind gift Dr. C. Cepko. We used PCR (polymerase chain reaction) to amplify out the dNhes1 cDNA from the Nolan-dNHes1 plasmid and cloned it into the CLPIT viral expression vector.

The transfections were performed in triplicate in a 6-well tissue culture dish format. A filler plasmid PBS KS PS was used to ensure that the total amount of DNA transfected in each well was 4 μg. 3 days after transfection, cells were lysed and analyzed for renilla and firefly luciferase activity using a luminometer (Turner Designs TD 20/20). The sensitivity was adjusted to ensure light levels remained within the instrument’s detection limits. The signal was integrated for 10 seconds with a 2 second delay and was reported in Relative Light Units (RLU).

The results were normalized to expression levels in the absence of any extrinsic transcription factors. In the presence of exogenous RBP-Jκ, the promoter activity decreased two-fold (Fig. S1). However, when NICD was added in addition to the RBP-Jκ, the promoter activity increased 2.5-fold above the basal level, implying the presence of a Notch pathway responsive element, such as the potential RBP-Jκ site. In the presence of exogenous Hes1, we observed a more than 10-fold decrease in the promoter activity, which was partially relieved by the addition of a dNHes1 construct, thus also confirming the presence of a functional N-box.

**Deterministic Model Equations**

(d Hcm)/dt = RfHcm/7-Hcm*kdHcm

(d Hcp)/dt = KtrHc*HcmD-kdHcp*Hcp-kniHcp*Hcp

(d Hnp)/dt = 7*kniHcp*Hcp-kdHnp*Hnp

Hdnp = Hnp^2^*KaHp

RfHcm = 1/D((1+4*Cr*Ka^2^*Kr^2^*NnpD^2^*RnpD^2^+2*Kr*RnpD*rR+2*Cr*Kr^2^*RnpD^2^*rR^2^+2*Ka*Kr*NnpD*RnpD*(1+2*Cr*Kr*RnpD*rR)+3*HdnpD*Kn*rNbox*(1+2*Cnr*Kr*RnpD*(Ka*NnpD+2*Cr*Ka^2^*Kr*NnpD^2^*RnpD+rR+2*Cr*Ka*Kr*NnpD*RnpD*rR+Cr*Kr*RnpD*rR^2^))+6*Cn*HdnpD^2^*Kn^2^*rNbox^2^*(1+2*Cnr*Kr*RnpD*(Ka*NnpD+2*Cr*Ka^2^*Kr*NnpD^2^*RnpD+rR+2*Cr*Ka*Kr*NnpD*RnpD*rR+Cr*Kr*RnpD*rR^2^))+6*Cn^2^*HdnpD^3^*Kn^3^*rNbox^3^*(1+2*Cnr*Kr*RnpD*(Ka*NnpD+2*Cr*Ka^2^*Kr*NnpD^2^*RnpD+rR+2*Cr*Ka*Kr*NnpD*RnpD*rR+Cr*Kr*RnpD*rR^2^)))*Vb+2*Ka*Kr*NnpD*(1+3*Cnr*HdnpD*Kn*rNbox*(1+2*Cn*HdnpD*Kn*rNbox+2*Cn^2^*HdnpD^2^*Kn^2^*rNbox^2^))*RnpD*(tc+2*Cr*Kr*RnpD*(Ka*NnpD+rR*tc))*Vmax)

Where, D = 1+2*Cr*Kr^2^*(1+2*Ka*NnpD+2*Ka^2^*NnpD^2^)*RnpD^2^+2*Kr*(RnpD+Ka*NnpD*RnpD)+3*HdnpD*Kn*(1+2*Cnr*Kr*RnpD*(1+Cr*Kr*RnpD+2*Cr*Ka^2^*Kr*NnpD^2^*RnpD+Ka*(NnpD+2*Cr*Kr*NnpD*RnpD)))+6*Cn*HdnpD^2^*Kn^2^*(1+2*Cnr*Kr*RnpD*(1+Cr*Kr*RnpD+2*Cr*Ka^2^*Kr*NnpD^2^*RnpD+Ka*(NnpD+2*Cr*Kr*NnpD*RnpD)))+6*Cn^2^*HdnpD^3^*Kn^3^*(1+2*Cnr*Kr*RnpD*(1+Cr*Kr*RnpD+2*Cr*Ka^2^*Kr*NnpD^2^*RnpD+Ka*(NnpD+2*Cr*Kr*NnpD*RnpD)))

(d Rcm)/dt = RfRcm/7-kdRcm*Rcm

(d Rcp)/dt = KtrRc*RcmD-kdRcp*Rcp-kniRcp*Rcp

(d Rnp)/dt = 7*kniRcp*Rcp-kdRnp*Rnp

RfRcm = 1/C((1+36*Cr^2^*Ka^3^*Kr^3^*NnpD^3^*RnpD^3^+3*Kr*RnpD*rR+6*Cr*Kr^2^*RnpD^2^*rR^2^+6*Cr^2^*Kr^3^*RnpD^3^*rR^3^+12*Cr*Ka^2^*Kr^2^*NnpD^2^*RnpD^2^*(1+3*Cr*Kr*RnpD*rR)+2*Ka*Kr*NnpD*RnpD*(1+3*Cr*Kr*RnpD*rR)^2^+3*HdnpD*Kn*rNbox*(1+3*Cnr*Kr*RnpD*(12*Cr^2^*Ka^3^*Kr^2^*NnpD^3^*RnpD^2^+4*Cr*Ka^2^*Kr*NnpD^2^*RnpD*(1+3*Cr*Kr*RnpD*rR)+rR*(1+2*Cr*Kr*RnpD*rR+2*Cr^2^*Kr^2^*RnpD^2^*rR^2^)+Ka*NnpD*(1+4*Cr*Kr*RnpD*rR+6*Cr^2^*Kr^2^*RnpD^2^*rR^2^)))+6*Cn*HdnpD^2^*Kn^2^*rNbox^2^*(1+3*Cnr*Kr*RnpD*(12*Cr^2^*Ka^3^*Kr^2^*NnpD^3^*RnpD^2^+4*Cr*Ka^2^*Kr*NnpD^2^*RnpD*(1+3*Cr*Kr*RnpD*rR)+rR*(1+2*Cr*Kr*RnpD*rR+2*Cr^2^*Kr^2^*RnpD^2^*rR^2^)+Ka*NnpD*(1+4*Cr*Kr*RnpD*rR+6*Cr^2^*Kr^2^*RnpD^2^*rR^2^)))+6*Cn^2^*HdnpD^3^*Kn^3^*rNbox^3^*(1+3*Cnr*Kr*RnpD*(12*Cr^2^*Ka^3^*Kr^2^*NnpD^3^*RnpD^2^+4*Cr*Ka^2^*Kr*NnpD^2^*RnpD*(1+3*Cr*Kr*RnpD*rR)+rR*(1+2*Cr*Kr*RnpD*rR+2*Cr^2^*Kr^2^*RnpD^2^*rR^2^)+Ka*NnpD*(1+4*Cr*Kr*RnpD*rR+6*Cr^2^*Kr^2^*RnpD^2^*rR^2^))))*Vbr+Ka*Kr*NnpD*RnpD*((2+9*Cnr*HdnpD*Kn*rNbox*(1+2*Cn*HdnpD*Kn*rNbox+2*Cn^2^*HdnpD^2^*Kn^2^*rNbox^2^))*tc^2^+12*Cr*Kr*(1+3*Cnr*HdnpD*Kn*rNbox*(1+2*Cn*HdnpD*Kn*rNbox+2*Cn^2^*HdnpD^2^*Kn^2^*rNbox^2^))*RnpD*tc*(Ka*NnpD+rR*tc)+18*Cr^2^*Kr^2^*(1+3*Cnr*HdnpD*Kn*rNbox*(1+2*Cn*HdnpD*Kn*rNbox+2*Cn^2^*HdnpD^2^*Kn^2^*rNbox^2^))*RnpD^2^*(2*Ka^2^*NnpD^2^+2*Ka*NnpD*rR*tc+rR^2^*tc^2^))*Vmaxr)

Where, C = 1+Kr*(3+2*Ka*NnpD)*RnpD+6*Cr*Kr^2^*(1+2*Ka*NnpD+2*Ka^2^*NnpD^2^)*RnpD^2^+6*Cr^2^*Kr^3^*(1+3*Ka*NnpD+6*Ka^2^*NnpD^2^+6*Ka^3^*NnpD^3^)*RnpD^3^+3*HdnpD*Kn*(1+3*Cnr*Kr*RnpD*(1+2*Cr*Kr*RnpD+2*Cr^2^*Kr^2^*RnpD^2^+12*Cr^2^*Ka^3^*Kr^2^*NnpD^3^*RnpD^2^+4*Cr*Ka^2^*Kr*NnpD^2^*RnpD*(1+3*Cr*Kr*RnpD)+Ka*NnpD*(1+4*Cr*Kr*RnpD+6*Cr^2^*Kr^2^*RnpD^2^)))+6*Cn*HdnpD^2^*Kn^2^*(1+3*Cnr*Kr*RnpD*(1+2*Cr*Kr*RnpD+2*Cr^2^*Kr^2^*RnpD^2^+12*Cr^2^*Ka^3^*Kr^2^*NnpD^3^*RnpD^2^+4*Cr*Ka^2^*Kr*NnpD^2^*RnpD*(1+3*Cr*Kr*RnpD)+Ka*NnpD*(1+4*Cr*Kr*RnpD+6*Cr^2^*Kr^2^*RnpD^2^)))+6*Cn^2^*HdnpD^3^*Kn^3^*(1+3*Cnr*Kr*RnpD*(1+2*Cr*Kr*RnpD+2*Cr^2^*Kr^2^*RnpD^2^+12*Cr^2^*Ka^3^*Kr^2^*NnpD^3^*RnpD^2^+4*Cr*Ka^2^*Kr*NnpD^2^*RnpD*(1+3*Cr*Kr*RnpD)+Ka*NnpD*(1+4*Cr*Kr*RnpD+6*Cr^2^*Kr^2^*RnpD^2^)))

(d Nm)/dt = RfNm/7-kdNm*Nm

(d Np)/dt = KtrN*NmD-kdNp*Np-Np*kDelp

(d Ncp)/dt = Np*kDelp-kniNcp*Ncp-kdNcp*Ncp

(d Nnp)/dt = 7*kniNcp*Ncp-kdNnp*Nnp

RfNm = 1/E(1+4*Cr*Ka^2^*Kr^2^*NnpD^2^*RnpD^2^+2*Kr*RnpD*rR+2*Cr*Kr^2^*RnpD^2^*rR^2^+2*Ka*Kr*NnpD*RnpD*(1+2*Cr*Kr*RnpD*rR)+HdnpD*Kn*rNbox*(1+2*Cnr*Kr*RnpD*(Ka*NnpD+2*Cr*Ka^2^*Kr*NnpD^2^*RnpD+rR+2*Cr*Ka*Kr*NnpD*RnpD*rR+Cr*Kr*RnpD*rR^2^)))*Vbn+2*Ka*Kr*NnpD*(1+Cnr*HdnpD*Kn*rNbox)*RnpD*(tc+2*Cr*Kr*RnpD*(Ka*NnpD+rR*tc))*Vmaxn)

Where, E = 1+2*Cr*Kr^2^*(1+2*Ka*NnpD+2*Ka^2^*NnpD^2^)*RnpD^2^+2*Kr*(RnpD+Ka*NnpD*RnpD)+HdnpD*Kn*(1+2*Cnr*Kr*RnpD*(1+Ka*NnpD+Cr*Kr*RnpD+2*Cr*Ka*Kr*NnpD*RnpD+2*Cr*Ka^2^*Kr*NnpD^2^*RnpD))

Each of the terms used in the above equations is explained in Table S1. All the parameters are listed in Table 1 of the manuscript.

**Table S1.** Explanation of each term in the deterministic model equations.

| **Symbol** | **Term in Equation** |
| --- | --- |
| Hcm | Hes1 cytoplasmic mRNA concentration |
| RfHcm | Rate of formation of Hes1 mRNA |
| Hcp | Hes1 cytoplasmic protein concentration |
| HcmD | Delayed cytoplasmic Hes1 mRNA concentration |
| Hnp | Hes1 nuclear protein concentration |
| Hdnp | Nuclear Hes1 dimer concentration |
| Rcm | RBP-Jκ cytoplasmic mRNA concentration |
| RfRcm | Rate of formation of RBP-Jκ mRNA |
| Rcp | RBP-Jκ cytoplasmic protein concentration |
| RcmD | Delayed cytoplasmic RBP-Jκ mRNA concentration |
| Rnp | RBP-Jκ nuclear protein concentration |
| Nm | Notch1 mRNA concentration |
| RfNm | Rate of formation of Notch1 mRNA |
| Np | Notch1 full-length protein concentration |
| NmD | Delayed Notch1 mRNA concentration |
| Ncp | Cytoplasmic NICD concentration |
| Nnp | Nuclear NICD concentration |
| HdnpD | Delayed Hes1 dimer concentration in nucleus |
| RnpD | Delayed nuclear RBP-Jκ protein concentration |
| NnpD | Delayed nuclear NICD concentration |
| C | Dummy variable used to denote the denominator of RfRcm |
| D | Dummy variable used to denote the denominator of RfHcm |
| E | Dummy variable used to denote the denominator of RfNm |

**Cytoplasm to nucleus dilution factor calculation**

The factor of 7 comes from the following calculation of the relative volumes of the cytoplasm and nucleus of a cell with a 5μm diameter nucleus and 10 μm diameter cell body:

$\frac{V_{Cytoplasm}}{V_{Nucleus}}=\frac{\frac{4}{3}\pi(5^{3}-2.5^{3})}{\frac{4}{3}\pi*2.5^{3}}=7$

Thus this dilution factor is applied whenever a molecule is transported between the nucleus and cytoplasm to take into account the change in concentration due to the change in volume.

**Time delay calculations**

The RNA polymerase II is estimated to process along DNA at a rate of approximately 20 nucleotides per second [5,6]. Also, from data from a study of two mammalian genes, it has been estimated that successive introns require between 0.4 to 7.5 min for splicing [5], and there is a further delay of approximately 4 min before the mature mRNA emerges into the cytosol [5]. The mRNA is translated by ribosomes progressing at approximately 6 nucleotides per second [7]. Based on these estimates, we arrive at the following numbers (Table S2) for T_m_ and T_p_ for the three genes.

**Table S2.** mRNA and protein time delays for the *hes1*, *RBP-Jk* and *notch1* genes.

| Gene | Transcript length | No. of Introns | Protein length | Tm (min) | Tp (min) |
| --- | --- | --- | --- | --- | --- |
| *hes1* | 2361 bp | 2 | 281 aa | 7<Tm<21 | 2.35 |
| *RBP-Jk* | 12254 bp | 11 | 515 aa | 18.6 < *T*_m_ < 96.7 | 4.3 |
| *notch1* | 56978 bp | 33 | 2518 aa | 64.7 < *T*_m_ < 299 | 21 |

**Transcription rate calculations**

We used 4.5 transcripts per minute [8] and 20 transcripts per minute [9] as initial estimates for *hes1* basal and maximum transcription rates respectively. The transcription rates for *RBP-Jκ*  and *notch1* were then determined from these estimates and the estimates of their minimum transcription times Thus, for *RBP-Jκ*, the basal rate is determined to be V_br_ = 4.5*(7/18.6) ~ 2 transcripts per minute, and for *notch1* it is V_bn_ = 4.5*(7/64.7) ~ 0.5 transcripts per minute. Similarly, the maximal transcription rate for *RBP-Jκ*, V_maxr_ = 20*(7/18.6) = 7.5 transcripts per minute, and for *notch1*, V_maxn_ = 20*(7/64.7) = 2.2 transcripts per minute.

**Hes1 repressive constant (r_Nbox_) estimation**

The repression due to 3 N-boxes is 40 fold [10]. Thus, given all other factors to be constant, rate of transcription with 3 hes1 bound to the r_Nbox_ in the promoter relative to the promoter without any hes1 bound is given by:

(r^3^_Nbox_*V_basal_ )/(V_basal_)= (1/40) ; Thus, rNbox =0.2924 ~ 0.3

The effect of each r_Nbox_ is multiplicative on the transcription rate.

**References**

1. Lewis AK, Frantz GD, Carpenter DA, de Sauvage FJ, Gao WQ (1998) Distinct expression patterns of notch family receptors and ligands during development of the mammalian inner ear. Mech Dev 78: 159-163.

2. Zhou S, Hayward SD (2001) Nuclear localization of CBF1 is regulated by interactions with the SMRT corepressor complex. Mol Cell Biol 21: 6222-6232.

3. Kopan R, Schroeter EH, Weintraub H, Nye JS (1996) Signal transduction by activated mNotch: importance of proteolytic processing and its regulation by the extracellular domain. Proc Natl Acad Sci U S A 93: 1683-1688.

4. Vooijs M, Schroeter EH, Pan Y, Blandford M, Kopan R (2004) Ectodomain shedding and intramembrane cleavage of mammalian Notch proteins is not regulated through oligomerization. J Biol Chem 279: 50864-50873.

5. Audibert A, Weil D, Dautry F (2002) In vivo kinetics of mRNA splicing and transport in mammalian cells. Mol Cell Biol 22: 6706-6718.

6. Ucker DS, Yamamoto KR (1984) Early events in the stimulation of mammary tumor virus RNA synthesis by glucocorticoids. Novel assays of transcription rates. J Biol Chem 259: 7416-7420.

7. Bruce Alberts AJ, Julian Lewis, Martin Raff, Keith Roberts, Peter Walter (2002) Molecular Biology of the Cell Garland Science, New York, NY.

8. Zeiser S, Muller J, Liebscher V (2007) Modeling the Hes1 oscillator. J Comput Biol 14: 984-1000.

9. Cinquin O (2007) Repressor dimerization in the zebrafish somitogenesis clock. PLoS Comput Biol 3: e32.

10. Takebayashi K, Sasai Y, Sakai Y, Watanabe T, Nakanishi S, et al. (1994) Structure, chromosomal locus, and promoter analysis of the gene encoding the mouse helix-loop-helix factor HES-1. Negative autoregulation through the multiple N box elements. J Biol Chem 269: 5150-5156.
