# Supplementary figures and images for: Computational Models of the Notch Network Elucidate Mechanisms of Context-dependent Signaling
Source: PLoS Comput Biol. 2009 May 22;5(5):e1000390. doi: 10.1371/journal.pcbi.1000390 (PMC2680760; doi:10.1371/journal.pcbi.1000390)

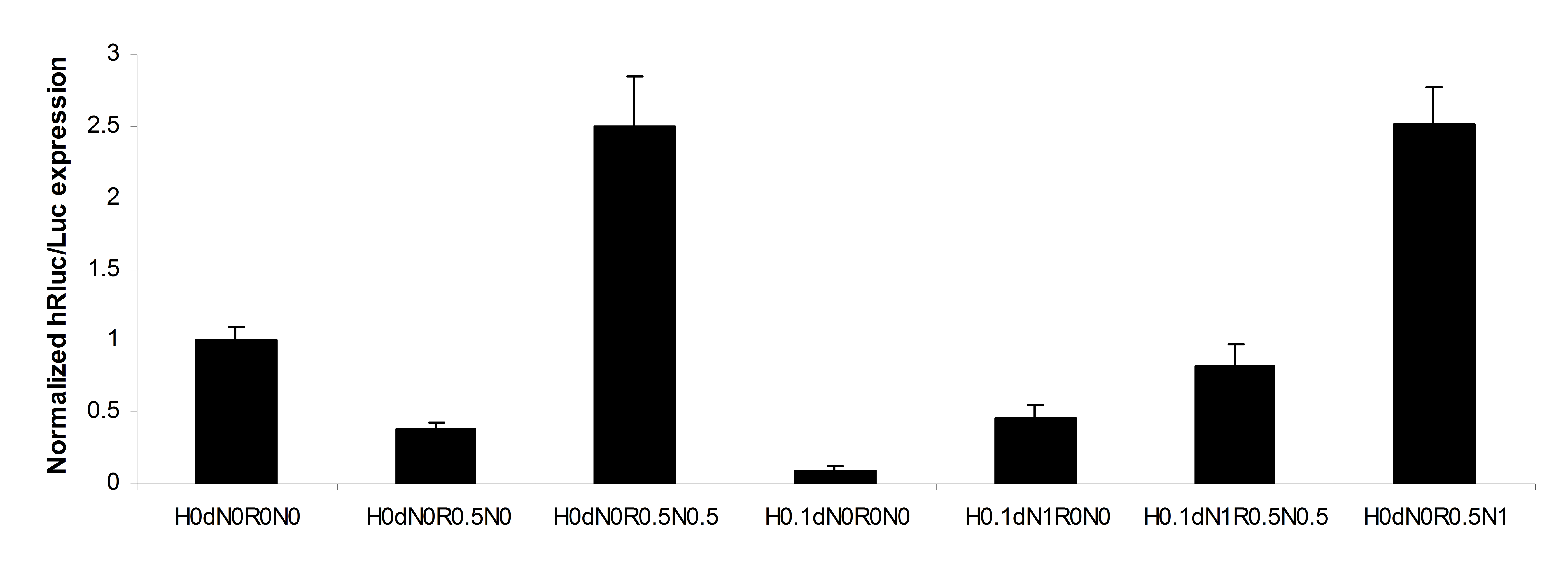

Supplement: Figure S1 — Transcriptional analysis of the Notch1 promoter. Relative fold changes in the activity of the Notch1 promoter in the presence of exogenous Hes1 (H), dNHes1 (dN), RBP-Jκ(R) and NICD (N) are shown. Relative amounts of plasmids used in each case encoding the respective cDNA are indicated. For example H0.1dN1R0.5N0.5 indicates Hes1 = 0.1 µg, dNHes1 = 1 µg, RBP-Jk = 0.5 µg and NICD = 0.5 µg in a total of 4 µg transfection. (2.52 MB TIF) [file pcbi.1000390.s002.tif]

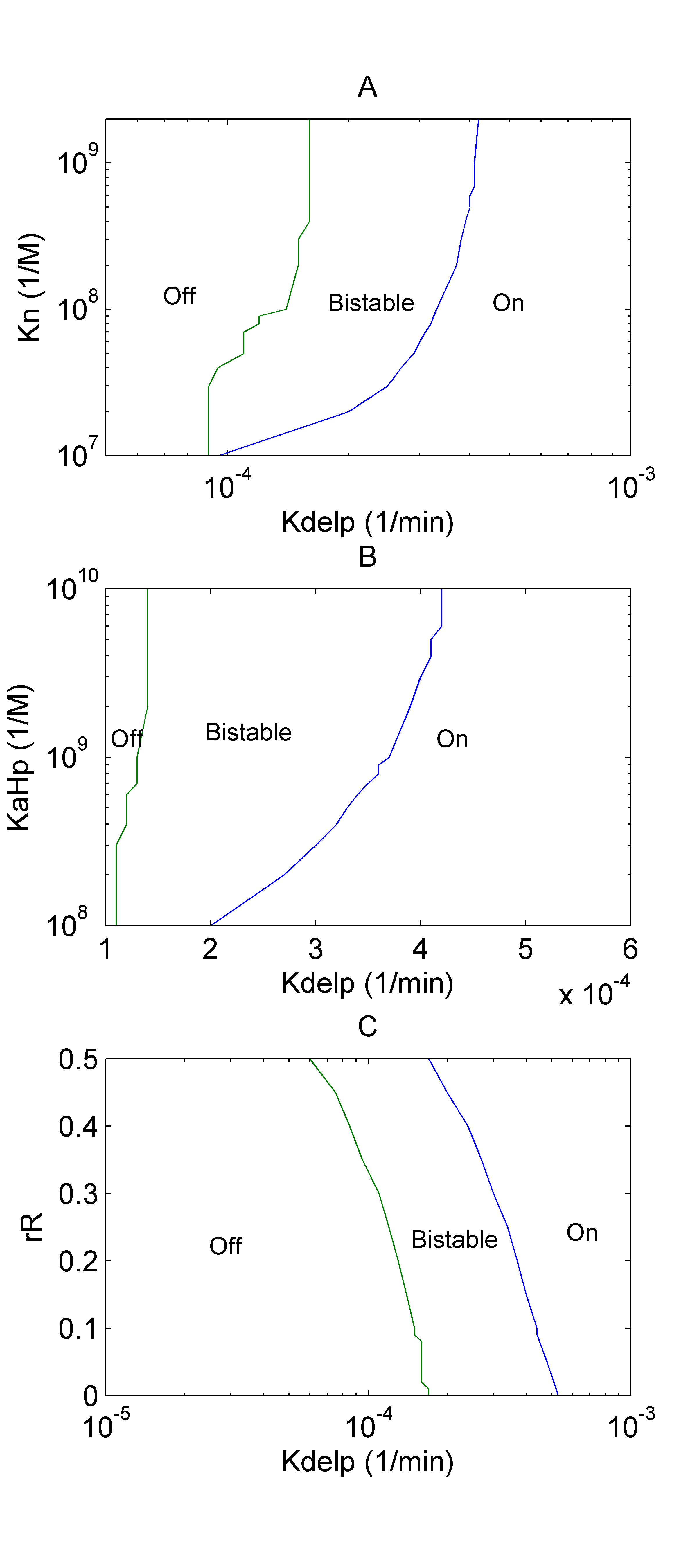

Supplement: Figure S2 — Bifurcation Analysis. (A) Bifurcation analysis of how the switching points vary with the Hes1 DNA association constant (Kn). Varying the association constant over two orders of magnitude causes a slight shift in the strength of the Delta signal required to switch the system state. Thus, stronger DNA association of Hes1 (higher values of Kn) increases the threshold values of Delta signal strength (Kdelp) required to turn the system ON. (B) Bifurcation analysis of how the switching points vary with the Hes1 dimerization constant (KaHp). Varying the dimerization constant over two orders of magnitude causes a slight increase in the strength of the Delta signal required to switch the system state to ON. (C) Bifurcation analysis of how the switching points vary with the repression constant of RBP-Jκ (rR). Increasing the repression constant from 0 to 0.5 (corresponding to a decrease in the RBP-Jκ repressive strength), has very little effect on the threshold of Delta signaling strength required to turn the system ON. (0.82 MB TIF) [file pcbi.1000390.s003.tif]

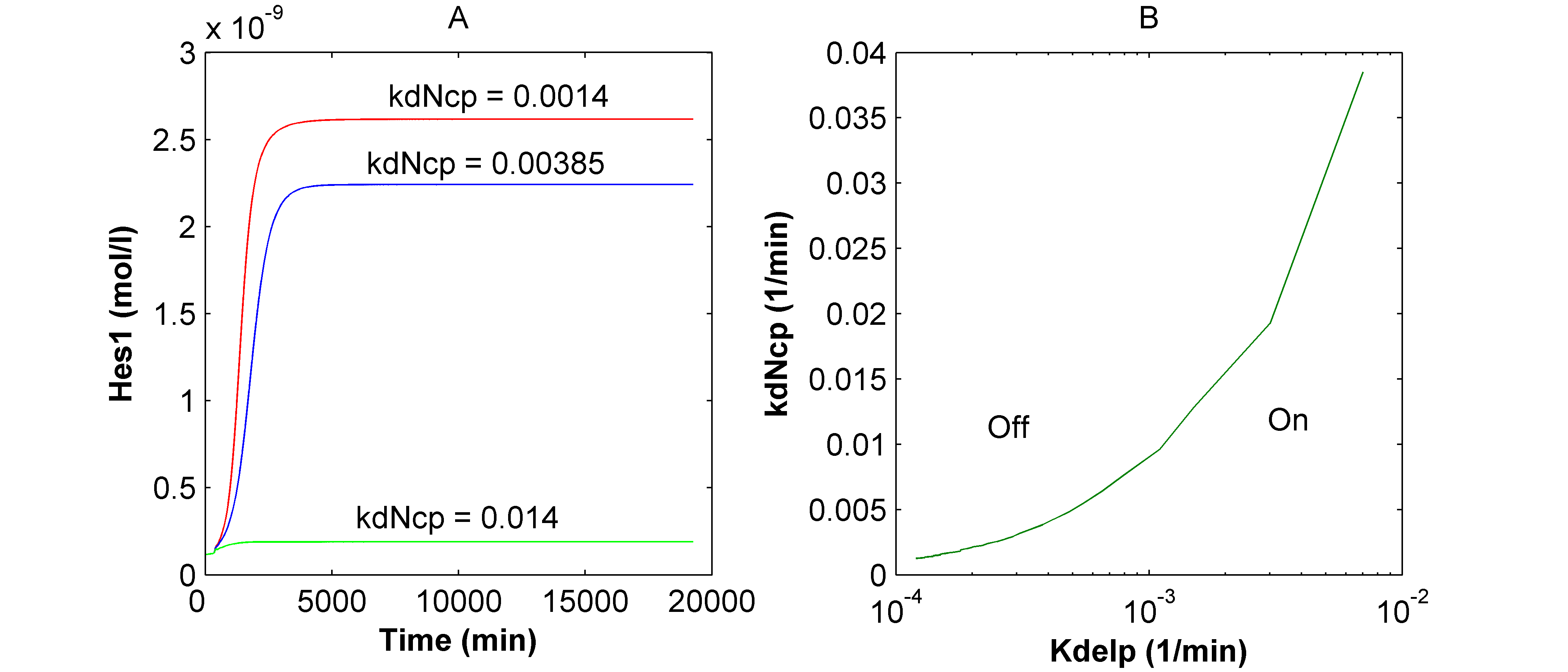

Supplement: Figure S3 — Effect of half-life of NICD on the Hes1 switch. (A) Changing the half life from 3 hrs (kdNcp = 0.00385) to 8 hrs (0.0014) due to the effect of GSK3β causes a slight increase in the steady state Hes1 concentration in response to a Delta signal of kDelp = 5×10^−4^, but no qualitative change in the switch. Increasing the degradation constant by 10-fold (kdNcp = 0.0014 to 0.014) however, causes complete suppression of the switch. (B) Analysis of how the threshold value of Delta signal required to switch the system from OFF to ON increases with the increasing degradation constant of NICD (decreasing NICD half life). (0.52 MB TIF) [file pcbi.1000390.s004.tif]

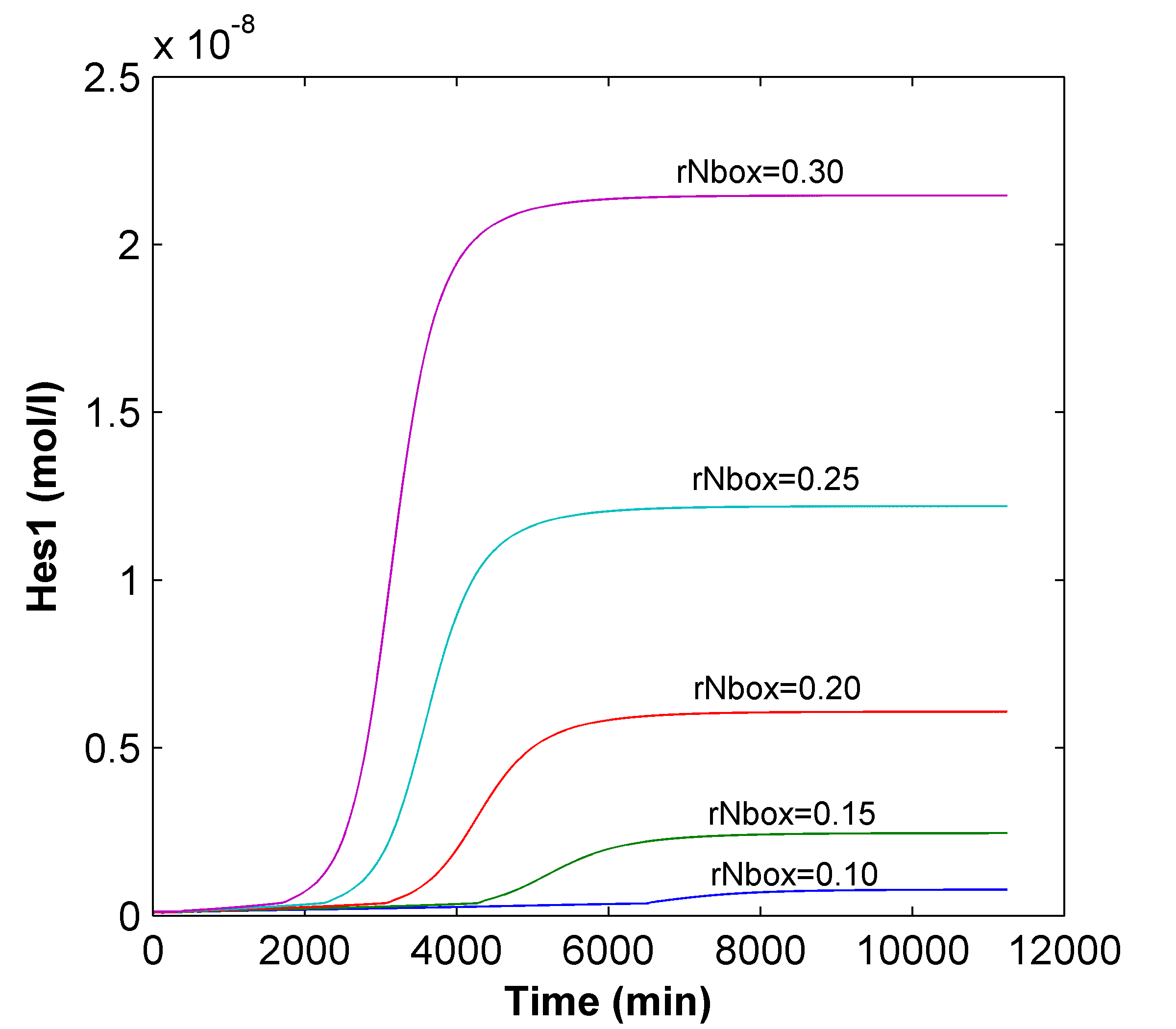

Supplement: Figure S4 — Effect of repression through Hes1 (rNbox) on the high steady state values of Hes1 expression.Decreasing rNbox progressively decreases the steady state concentrations of Hes1 in the rNbox range of 0.3 to 0.1. (0.42 MB TIF) [file pcbi.1000390.s005.tif]
